# Enteroendocrine signaling in irritable bowel syndrome: beyond cell counts toward stimulus-responsive physiological profiling

**DOI:** 10.3389/fmed.2026.1992567

**Published:** 2026-10-23

**Authors:** Jiayi Han, Yixuan Lang, Kangmeng Yao, Jia Mi

**Affiliations:** 1 College of Traditional Chinese Medicine, Changchun University of Chinese Medicine, Changchun, Jilin, China; 2 The Affiliated Hospital of Changchun University of Chinese Medicine, Changchun, Jilin, China

**Keywords:** enteroendocrine cells, gut hormones, irritable bowel syndrome, meal challenge, nutrient sensing, serotonin, visceral hypersensitivity

## Abstract

Meals are a major symptom trigger in irritable bowel syndrome (IBS), yet the endocrine steps linking luminal nutrients to pain, bloating, urgency, and altered bowel habits remain poorly defined. Early studies relied heavily on mucosal enteroendocrine cell (EEC) counts. Those observations remain informative, particularly in post-infectious IBS, but findings vary with intestinal site, bowel-habit phenotype, staining, and morphometry, and cell abundance does not measure secretory function. Human meal-challenge studies distinguish circulating concentrations from stimulus-evoked responses. In IBS with diarrhea, higher fasting and fed plasma 5-hydroxytryptamine (serotonin, 5-HT) may reflect altered handling more than a uniformly exaggerated postprandial response, whereas IBS with constipation can show impaired stimulus-evoked release. Cholecystokinin, peptide YY, motilin, ghrelin, and related peptides also vary with sex, bowel habit, and visceral sensitivity. Recent human organoid and transcriptomic studies identify distinct nutrient-sensing programs among EEC subtypes and provide a cellular framework for this heterogeneity. We integrate these findings across luminal sensing, secretion, metabolism, receptor responsiveness, and motor or sensory output. The evidence does not support a single IBS endocrine signature. Standardized nutrient challenges linked to serial hormone measurements, mucosal molecular data, and objective physiological readouts are more likely to yield reproducible and clinically testable response patterns.

## 1 Introduction

Irritable bowel syndrome (IBS) is a disorder of gut–brain interaction defined by recurrent abdominal pain associated with altered bowel habits (1–3). Meals are among the most frequently reported triggers. In a well-characterized clinical cohort, food-related gastrointestinal symptoms were common and were associated with more severe IBS and poorer quality of life (4). The biological explanation is unlikely to be a single pathway. Luminal distension, maldigestion, visceral hypersensitivity, mucosal immune activation, microbial metabolites, altered motility, and central processing can all contribute, often in the same patient (3, 5, 6).

Enteroendocrine cells (EECs) occupy a strategic position within this network. They sense nutrients and microbial products at the luminal surface and release mediators such as 5-hydroxytryptamine (serotonin, 5-HT), cholecystokinin (CCK), peptide YY (PYY), glucagon-like peptide-1 (GLP-1), glucose-dependent insulinotropic polypeptide, and ghrelin. Their products influence secretion, motility, satiety, epithelial function, and visceral signaling (7, 8). Human organoid and single-cell work has also made clear that EECs are not a uniform population: receptor expression and secretory programs vary by cell type and intestinal region (9, 10). Some EECs form close functional links with neural circuits, adding a rapid route from luminal sensing to the gut–brain axis (11).

A 2014 review drew attention to interactions between ingested nutrients and gut endocrine cells in IBS (12). Subsequent studies have refined this framework. More rigorous morphometry has questioned a universal cell-number abnormality, meal studies have separated fasting concentrations from stimulus-evoked responses, and human organoids now allow direct study of nutrient and microbial sensing. Recent reviews have covered broad IBS pathophysiology, food-triggered pain, and microbiome–EEC communication (3, 6, 11, 13). The narrower question addressed here is how these newer cellular observations relate to older human biopsy and challenge studies. Rather than listing hormone abnormalities, this Review examines the sequence from luminal detection to secretion, hormone handling, receptor response, physiological output, and symptoms.

An EEC response is not a single measurable event. Nutrient delivery to a given intestinal region, the receptor repertoire of the local EEC population, intracellular coupling and exocytosis, hormone uptake or degradation, target-receptor sensitivity, and downstream neural or motor activity all influence the final observation. Similar circulating concentrations may therefore arise from different cellular processes, while a biologically important local signal may produce little change in peripheral blood. Here, stimulus-responsive physiological profiling refers to coordinated measurement across more than one of these levels; it is a research framework, not a proposed clinical test (7–11).

## 2 Literature search and synthesis

PubMed/MEDLINE was searched from database inception to 2 September 2026 using combinations of the terms irritable bowel syndrome, enteroendocrine cell, enterochromaffin cell, serotonin, gut hormone, meal challenge, nutrient challenge, cholecystokinin, peptide YY, motilin, GLP-1, nutrient sensing, chemosensitivity, bile acid, organoid, and visceral hypersensitivity. Reference lists and citing articles of key studies were checked for additional reports. Priority was given to English-language adult human studies that directly examined EEC abundance, meal- or nutrient-evoked hormone responses, intestinal chemosensing, or pharmacological modulation of relevant pathways. Recent human organoid and single-cell studies were included to provide mechanistic context. This was a focused narrative Review; study selection was purposive, and no formal systematic screening or risk-of-bias assessment was performed.

ChatGPT was used during manuscript preparation to organize literature notes, assist with preliminary drafting and language revision, check reference consistency, and plan the initial conceptual-figure layout. It was not used as a source of scientific evidence or for data analysis. Product details and the scope of use are reported in the Acknowledgments.

## 3 What static biopsies can—and cannot—show

The earliest evidence for an EEC abnormality was strongest in post-infectious IBS (PI-IBS). After Campylobacter enteritis, rectal EEC expansion can persist alongside mucosal lymphocytosis and increased permeability (14). Patients who go on to develop PI-IBS have also shown greater enterochromaffin cell hyperplasia than post-infectious controls, although anxiety and depression contributed independently to clinical outcome (15). Other investigators reported lower densities of selected duodenal endocrine populations, including CCK- and secretin-immunoreactive cells, particularly in IBS with diarrhea (IBS-D) (16). These findings support mucosal endocrine remodeling in defined contexts, but not a single histological pattern across IBS.

Several studies found no overall difference in EEC abundance. Park and colleagues compared 25 patients with IBS-D and 13 controls and found similar chromogranin A-positive cell counts in the terminal ileum, ascending colon, and rectum. Within the combined sample, however, rectal EEC counts were inversely related to the maximally tolerated rectal pressure, and hypersensitive patients had higher rectal counts than normosensitive participants (17). The association was modest, but it suggested that cell abundance might track a physiological trait more closely than the diagnostic label.

Morphometry can itself create disagreement. Mohammadian and colleagues counted jejunal 5-HT-positive and chromogranin A-positive cells both per high-power field and per square millimeter of mucosal area. IBS and control groups did not differ overall, while high-power-field counting systematically underestimated the number of positive cells compared with area-based analysis (18). Intestinal site, biopsy orientation, staining protocol, denominator, and cohort composition all affect the result. More fundamentally, a count is a snapshot. Similar numbers of EECs can coexist with different receptor expression, excitability, secretion, local hormone metabolism, or neural coupling.

Single-cell studies also show why a stable total EEC count can be misleading. Chromogranin A-positive cells include several hormone-defined populations with different regional distributions and sensing machinery. Human duodenal and ileal organoids contain distinct EEC clusters, and receptors for bile acids, long- and short-chain fatty acids, and glucose are not expressed uniformly across them (9). A biopsy could therefore show an unchanged total count while the relative abundance or molecular state of particular EEC subsets differs. No human IBS study has yet combined quantitative histology, cell-type-resolved transcriptomics, and stimulus-evoked secretion in the same participants. Until such studies are available, broad immunostaining should be interpreted as anatomical context rather than a direct measure of endocrine capacity.

## 4 Meal- and nutrient-evoked endocrine responses

### 4.1 5-HT: release, availability, and handling

5-HT provides the clearest human example of why fasting concentration, postprandial concentration, and change from baseline cannot be treated as the same endpoint. Dunlop and colleagues studied 15 patients with PI-IBS, 15 with IBS with constipation (IBS-C), and 15 controls after a standardized 522-kcal meal. Postprandial plasma 5-HT exposure was markedly lower in IBS-C, whereas PI-IBS showed higher peak concentrations. Rectal enterochromaffin cell counts were similar across groups, but mucosal 5-hydroxyindoleacetic acid (5-HIAA)/5-HT ratios were reduced in both IBS phenotypes (19). Because 5-HIAA is the major oxidative metabolite of 5-HT, the 5-HIAA/5-HT ratio can serve as an index of mucosal 5-HT turnover; a lower ratio is compatible with reduced local uptake or metabolism, rather than directly indicating fewer cells or impaired stimulus-evoked secretion. Functional serotonergic abnormalities were therefore present without a clear numerical enterochromaffin-cell difference.

Atkinson and colleagues extended the comparison to 55 patients with IBS-D, 29 with IBS-C, and 35 controls. IBS-D was characterized by higher fasting and fed platelet-depleted plasma 5-HT, but the meal-induced increase relative to baseline was similar to that in controls. IBS-C showed little or no postprandial increase and lower fed exposure and peak concentrations (20). Together with 5-HIAA and platelet 5-HT measurements, the authors interpreted the pattern as reduced reuptake or altered handling in IBS-D and impaired stimulus-evoked release in IBS-C. An earlier study in symptomatic women with IBS-D also found higher fed plasma 5-HT (21).

Hormone concentration did not map neatly onto symptoms. Both PI-IBS and IBS-C participants in the Dunlop study developed postprandial symptoms despite opposite 5-HT response patterns, and plasma exposure was not correlated with pain, nausea, bloating, or fullness (19). Atkinson and colleagues likewise recorded similar meal-related symptom worsening in IBS-C and IBS-D despite divergent serotonergic profiles (20). 5-HT may shape motility, secretion, bowel habit, receptor sensitivity, and reflex signaling without behaving as a simple concentration-dependent pain mediator.

### 4.2 CCK, motilin, PYY, ghrelin, and integrated physiological readouts

Other peptides show the same dependence on stimulus and phenotype. In an early fat-meal study, patients with IBS had reduced motilin release but a more prolonged CCK response than controls (22). A larger study by van der Veek and colleagues measured CCK, motilin, and PYY in 99 patients and 40 controls after an 800-kcal meal. Fasting CCK and the incremental postprandial CCK response were higher in IBS overall, whereas mean motilin and PYY responses did not differ. Subgroup analysis was more informative: fasting motilin was higher in IBS-D than in IBS-C or alternating IBS, postprandial PYY was greater in patients with visceral hypersensitivity, and CCK differences were most evident in women (23).

Furgała and colleagues brought endocrine and physiological measurements into the same experiment. Forty-two Rome IV-defined IBS-C patients and 42 matched controls underwent measurement of gastrin, CCK, vasoactive intestinal peptide, ghrelin, insulin, and multichannel electrogastrography before and after a 300-kcal meal. The IBS-C group showed altered fasting and postprandial hormone profiles together with less normogastria, more dysrhythmia, and a blunted postprandial electrical response (24). Electrogastrography is an indirect measure of gastric motor function. In IBS-C, postprandial gastrin correlated negatively with normogastria, dominant power, and slow-wave coupling, and insulin correlated negatively with bradygastria (24). These associations do not establish causality because the observational design did not manipulate hormone signaling. Even so, the design illustrates what is missing from many earlier studies: endocrine data are easier to interpret when a motor or sensory output is measured at the same time.

Table 1 summarizes representative human studies that shaped the distinction between a static biopsy result and a stimulus-responsive endocrine profile.

**Table 1:** Representative human studies informing the shift from static EEC measurements to stimulus-responsive endocrine profiling in IBS.

Study/phenotype	Sample size	Rome criteria	Assessment	Main finding	Interpretive boundary
Spiller et al. (2000) (14) Post-dysenteric/PI-IBS	21 post-Campylobacter patients; 10 PD-IBS; 12 controls	Rome I	Rectal biopsy; EECs, T cells, permeability	Persistent rectal EEC expansion and immune changes after Campylobacter supported a defined post-infectious mucosal phenotype.	Post-infectious context; histology does not measure stimulus-evoked secretion.
Dunlop et al. (2005) (19) PI-IBS/IBS-C	15 PI-IBS; 15 IBS-C; 15 controls	Rome II	522-kcal meal; plasma 5-HT; rectal EC counts and mucosal 5-HT/5-HIAA; colonic transit	IBS-C had lower postprandial 5-HT exposure, whereas PI-IBS had higher peak 5-HT despite similar EC counts.	Small groups; systemic 5-HT did not correlate simply with postprandial symptoms.
Atkinson et al. (2006) (20) IBS-D/IBS-C	55 IBS-D; 29 IBS-C; 35 controls	Rome II	Standard meal; fasting and fed plasma 5-HT/5-HIAA; platelet 5-HT	IBS-D had high fasting and fed 5-HT but a baseline-adjusted response similar to controls; IBS-C showed little meal-induced rise.	Supports altered handling versus impaired release; findings depend on the chosen endpoint.
Park et al. (2006) (17) IBS-D	25 IBS-D; 13 controls	Rome II	Ileal, colonic, and rectal biopsies; chromogranin A; rectal barostat	No overall count difference; rectal EEC abundance was associated with lower maximal tolerable pressure.	Modest association in a small cohort; chromogranin A is a broad EEC marker.
van der Veek et al. (2006) (23) IBS subtypes	99 IBS; 40 controls	Rome II	800-kcal meal; CCK, motilin, PYY; rectal barostat	CCK was higher overall; fasting motilin varied by bowel habit and PYY response by visceral sensitivity.	Subgroup signals were not uniform across peptides and require replication.
Mohammadian et al. (2020) (18) IBS subtypes and controls	51 IBS; 35 controls	Rome II	Jejunal biopsy; 5-HT and chromogranin A; two morphometric methods	No overall EEC difference; high-power-field counting underestimated abundance compared with area-based quantification.	Demonstrates method dependence; similar counts do not establish similar function.
Grover et al. (2021) (27) IBS-C/IBS-D	12 IBS-C; 14 IBS-D; 15 controls	Rome III	Small-bowel lipid challenge; barostat; pCLE; duodenal/jejunal RNA-seq	Lipid evoked more symptoms; TRPV expression was associated with symptoms and sensory thresholds.	Bulk RNA-seq cannot identify the cellular source of TRPV1/TRPV3 expression; hormones were not measured concurrently.
Furgała et al. (2023) (24) IBS-C	42 IBS-C; 42 controls	Rome IV	300-kcal meal; multiple gut peptides; multichannel electrogastrography	Altered hormone profiles accompanied impaired gastric electrical adaptation.	Restricted to IBS-C; electrogastrography is an indirect motor measure.
Khan et al. (2025) (40) IBS-D/IBS-M	30 IBS-D/IBS-M; no control group	Rome IV	12-week low-FODMAP intervention; plasma GLP-1; IBS-SSS	GLP-1 increased while symptoms and body weight decreased.	Uncontrolled intervention; no standardized meal challenge or mediation analysis.

CCK, cholecystokinin; EC, enterochromaffin cell; EEC, enteroendocrine cell; EGG, electrogastrography; IBS-C, constipation-predominant IBS; IBS-D, diarrhea-predominant IBS; IBS-M, mixed IBS; PI-IBS, post-infectious IBS; pCLE, probe-based confocal laser endomicroscopy; PYY, peptide YY; 5-HIAA, 5-hydroxyindoleacetic acid; 5-HT, 5-hydroxytryptamine. The table is illustrative rather than exhaustive. Additional abbreviations: GLP-1, glucagon-like peptide-1; IBS, irritable bowel syndrome; IBS-SSS, IBS Severity Scoring System; FODMAP, fermentable oligosaccharides, disaccharides, monosaccharides, and polyols; RNA-seq, RNA sequencing; TRPV, transient receptor potential vanilloid. Sample sizes denote study groups; outcome-specific denominators can differ (Khan et al.: GLP-1, *n* = 30; IBS-SSS, *n* = 29). PD-IBS in the Spiller cohort refers to post-dysenteric IBS.

## 5 Nutrient sensing upstream of hormone release

### 5.1 Receptor repertoires and stimulus–secretion coupling

Meal-evoked endocrine responses are embedded in a broader chemosensory system. Controlled nutrient experiments show that the composition of a stimulus matters. Lipid delivery can increase rectal or colonic sensitivity in IBS, with effects that vary by bowel habit, sex, and psychological factors (25, 26). These studies were not designed to isolate EECs, but they establish the physiological setting in which EEC-derived signals are released and interpreted.

Grover and colleagues combined small-bowel lipid infusion with rectal barostat testing, confocal endomicroscopy, and duodenal and jejunal transcriptomics in 26 patients with IBS and 15 healthy volunteers. Lipid exposure produced more pain, bloating, nausea, fullness, and urgency in IBS. Baseline permeability and overall mucosal architectural responses did not clearly separate the groups. By contrast, duodenal transient receptor potential vanilloid 3 (TRPV3) expression was increased in both IBS-C and IBS-D, and TRPV1 was increased in IBS-D; higher expression was associated with lipid-evoked symptoms and lower sensory thresholds (27). Because TRPV1 and TRPV3 expression was measured by bulk RNA sequencing of mucosal biopsies, the expressing cell types could not be identified; gut hormones were not measured concurrently. The study therefore supports altered small-intestinal chemosensitivity, not an EEC-specific TRPV mechanism.

Human organoid studies provide a cellular framework for such heterogeneity. EEC subtypes differ in expression of G protein-coupled bile acid receptor 1 (GPBAR1, also known as TGR5), free fatty acid receptors 1 (FFAR1, also known as GPR40) and 2 (FFAR2), the sodium/glucose cotransporter 1 (SLC5A1, also known as SGLT1), and other nutrient-sensing machinery (9). Human enterochromaffin cells respond experimentally to bacterial metabolites, aromatic amino acids, and adrenergic inputs through a diverse receptor repertoire (10). Luminal nutrients and microbial products can therefore modify secretion through several routes (28, 29, 34), while food antigen exposure may activate local immune pathways that sensitize visceral afferents independently of a measurable endocrine response (6, 30).

The recent human EEC atlas makes the first step of this chain more concrete. In organoid-derived cells, GPBAR1 expression was greatest in GLP-1/PYY-producing L cells; FFAR1 was enriched in I, K, and L cells; FFAR2 was prominent in ileal L cells and enterochromaffin cells; and SLC5A1 was highly expressed in K and L cells (9). This distribution predicts that the same luminal mixture will not be sensed identically along the intestine or by every EEC subtype. It also cautions against assigning a circulating hormone change to a nonspecific increase or decrease in ‘EEC activity.’ The measured response depends on which cells are exposed, which receptors they express, and whether those receptors are coupled to secretion under the experimental conditions.

Functional experiments in labelled human enterochromaffin organoids extend the point beyond transcript abundance. These cells expressed receptors for nutrients, microbial metabolites, gut hormones, and adrenergic signals, and responded to selected bacterial metabolites, aromatic amino acids, and adrenergic agonists through calcium, cyclic-AMP, electrical, or 5-HT secretory responses (10). Receptor expression alone was therefore not equivalent to a functional output, and different stimuli engaged different intracellular readouts. Applied to IBS, this suggests several testable sites of divergence—receptor abundance, second-messenger coupling, excitability, vesicle release, or paracrine regulation—but none has yet been mapped directly onto the meal-response patterns described in clinical cohorts.

Bile acids illustrate how a luminal signal can connect endocrine sensing to transit. IBS-D is not synonymous with bile acid diarrhea, but a subgroup has increased bile acid synthesis or fecal excretion, measurable with serum 7*α*-hydroxy-4-cholesten-3-one and fecal bile acids, with fibroblast growth factor 19 providing complementary information on feedback regulation (31). Genetic variation in GPBAR1 has also been associated with quantitative differences in colonic transit and fecal bile acid excretion (32). These observations make a bile acid–EEC–motor pathway plausible, although human IBS studies have rarely combined bile acid phenotyping with meal-evoked hormone measurements.

### 5.2 From local secretion to measured physiological output

Blood concentrations depend on more than secretion. For 5-HT, mucosal cells take up and metabolize the released mediator, while portal and hepatic handling and platelet uptake further shape the platelet-depleted plasma concentration measured in challenge studies (19, 20, 33). Other EEC products may act locally on epithelial, immune, neural, or vascular targets before appreciable systemic exposure (8, 11). An unchanged peripheral concentration does not exclude altered local or downstream signaling. For example, oral 5-hydroxytryptophan increased rectal pain sensitivity in hypersensitive IBS patients without a detectable change in plasma 5-HT (43). This demonstrates a dissociation between circulating 5-HT and the sensory response, although it does not establish the site of serotonergic action. A high fed concentration may instead reflect an elevated baseline or slower clearance rather than excessive stimulus-evoked release.

This distinction changes what can be inferred from a result. A study that measures only circulating hormone cannot localize an abnormality to sensing, secretion, clearance, or target responsiveness. Conversely, a mucosal transcript or receptor measurement does not show that secretion occurs during a meal. To identify where the pathway diverges, experiments should link a defined stimulus to serial hormone values, local molecular measurements, and a time-matched motor or sensory endpoint. The objective is not to maximize the number of assays, but to choose measurements that distinguish competing mechanisms.

### 5.3 Organoid-based selection and testing of candidate stimuli

Organoids can help select a human challenge, rather than serving only to explain its results afterward. A first tier should characterize region-matched, donor-derived cultures for total EEC abundance, hormone-defined subtype proportions, receptor expression, and basal hormone content. The human EEC atlas provides starting hypotheses for glucose, lipid, bile acid, and microbial-metabolite challenges, but transcript abundance alone does not establish stimulus–secretion coupling (9, 10). Figures 1, 2 link the profiling framework and selected sensing pathways to candidate stimuli and paired physiological measurements. These mappings guide experimental selection; they do not establish an IBS-specific receptor profile.

**Figure 1:**
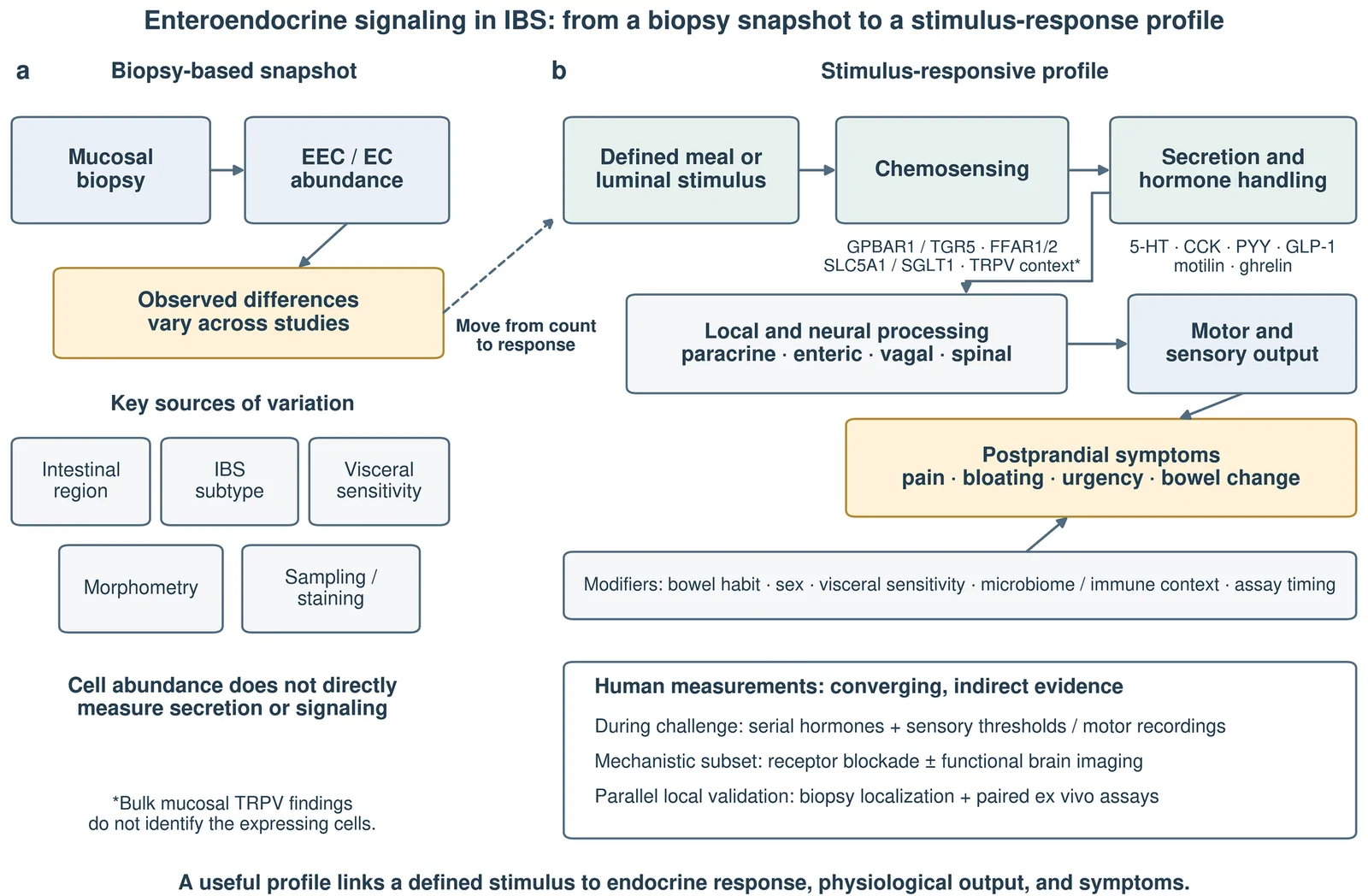
From a biopsy snapshot to a stimulus-responsive enteroendocrine profile in IBS. The left panel summarizes the limits of interpreting mucosal EEC abundance without accounting for intestinal region, IBS subtype, visceral sensitivity, and morphometric method. The right panel follows a defined meal or luminal stimulus through chemosensing, EEC secretion and hormone handling, local and neural processing, motor or sensory output, and postprandial symptoms. Human measurements can combine site-matched mucosal assays with challenge-linked sensory or motor recordings and, in selected mechanistic studies, receptor blockade and functional brain imaging. Mucosal and *ex vivo* assays provide local mechanistic context; sensory recordings and imaging infer downstream processing and do not directly demonstrate EEC-to-nerve transmission (see Section 7.3). GPBAR1, FFAR1/2, SLC5A1, and TRPV channels provide mechanistic context from human EEC and small-intestinal chemosensory studies; the TRPV findings are not assumed to be EEC-specific. The proposed profile is a research framework rather than a validated clinical test. CCK, cholecystokinin; EC, enterochromaffin cell; EEC, enteroendocrine cell; FFAR1/2, free fatty acid receptors 1 and 2; fMRI, functional magnetic resonance imaging; GLP-1, glucagon-like peptide-1; GPBAR1/TGR5, G protein-coupled bile acid receptor 1/Takeda G protein-coupled receptor 5; IBS, irritable bowel syndrome; PYY, peptide YY; SLC5A1/SGLT1, solute carrier family 5 member 1/sodium–glucose cotransporter 1; TRPV, transient receptor potential vanilloid; 5-HT, 5-hydroxytryptamine.

**Figure 2:**
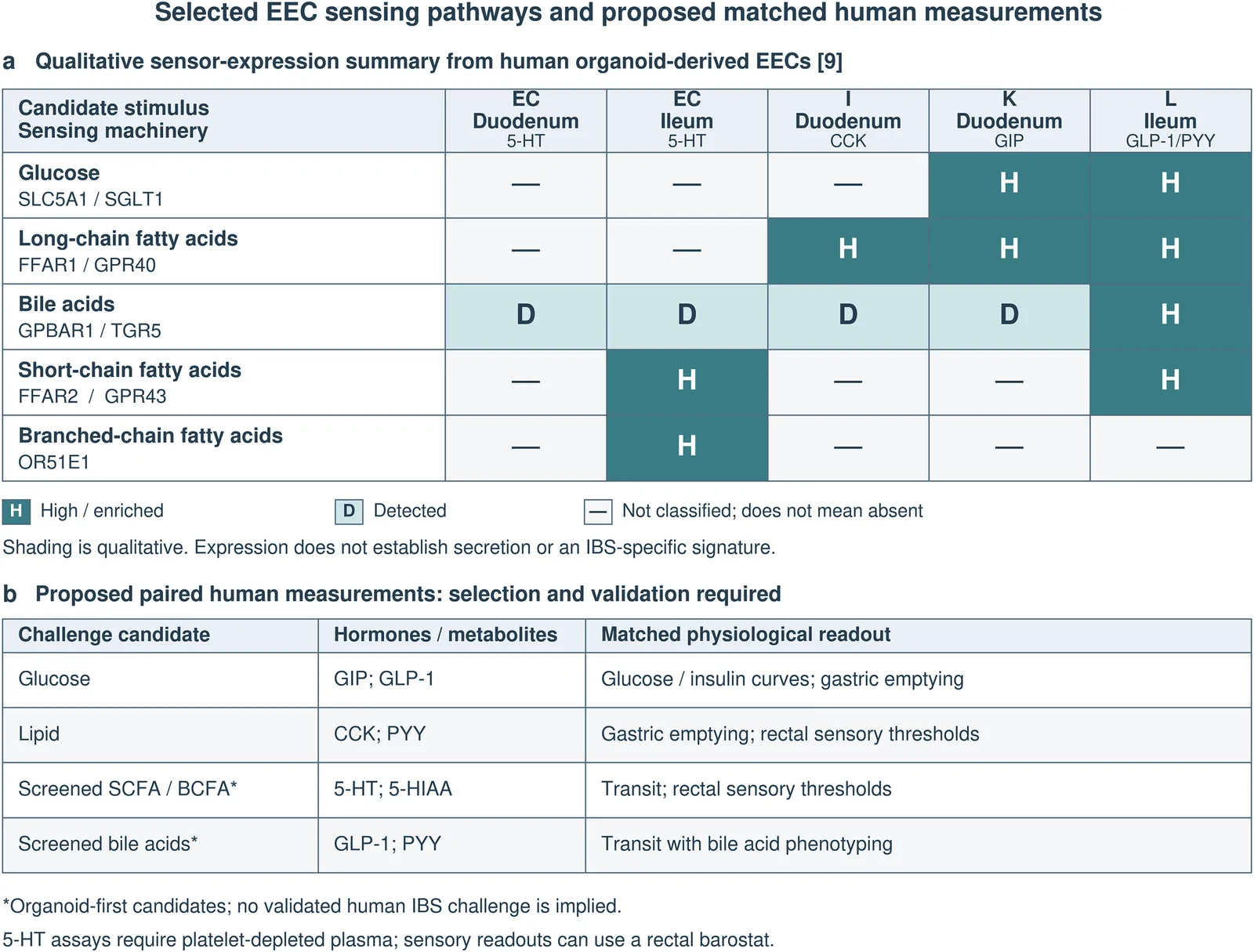
Candidate stimuli, EEC sensing machinery, and paired physiological measurements. The matrix qualitatively summarizes selected receptor or transporter expression patterns reported in human duodenal and ileal organoid-derived EECs (9). H denotes reported high or enriched expression; D denotes reported detected expression without selected enrichment; a dash indicates that a cell–sensor combination is not classified here, rather than receptor absence. Shading does not reproduce quantitative expression values, demonstrate stimulus-evoked secretion, or establish an IBS-specific signature. EEC labels identify hormone-enriched clusters and do not exclude hormone coexpression. The lower panel presents proposed pairings of serial hormone assays with physiological measurements; bile acid and microbial-metabolite candidates require organoid screening before human challenge selection (10, 19, 20, 23, 24, 27, 31, 32). IBS, irritable bowel syndrome; EC, enterochromaffin cell; EEC, enteroendocrine cell; I, CCK-enriched EEC; K, GIP-enriched EEC; L, GLP-1/PYY-enriched EEC; 5-HT, 5-hydroxytryptamine (serotonin); 5-HIAA, 5-hydroxyindoleacetic acid; CCK, cholecystokinin; GIP, glucose-dependent insulinotropic polypeptide; GLP-1, glucagon-like peptide-1; PYY, peptide YY; SLC5A1/SGLT1, solute carrier family 5 member 1/sodium–glucose cotransporter 1; FFAR1/GPR40, free fatty acid receptor 1/G protein-coupled receptor 40; FFAR2/GPR43, free fatty acid receptor 2/G protein-coupled receptor 43; GPBAR1/TGR5, G protein-coupled bile acid receptor 1/Takeda G protein-coupled receptor 5; OR51E1, olfactory receptor family 51 subfamily E member 1; SCFA, short-chain fatty acid; BCFA, branched-chain fatty acid. The matrix is selective rather than exhaustive; it does not assign motilin or ghrelin responses from receptor expression alone.

A second tier should screen concentration–response relationships in organoid-derived monolayers on permeable supports. These cultures permit controlled apical exposure, separate basolateral hormone collection, and simultaneous measurement of transepithelial electrical resistance or tracer permeability (44, 45). Barrier measurements help distinguish regulated secretion from leakage or epithelial injury. Apical and basolateral stimulation should be compared where receptor location is relevant, particularly for bile acid sensing. By contrast, conventional enclosed 3D organoids retain epithelial architecture but restrict access to the apical lumen; bath application mainly exposes the outer, basolateral surface unless the lumen is accessed by microinjection or polarity is modified (45). The formats therefore answer complementary questions. Dose ranges should approximate plausible regional exposures, with vehicle, osmolarity, pH, viability, and secretory-capacity controls. Calcium or cyclic-AMP responses can support interpretation, while receptor inhibition or genetic perturbation can test whether a candidate sensor contributes to hormone release (10, 47).

The denominator must remain explicit. Secretion per well measures total culture output; secretion per milligram of protein adjusts for culture biomass; neither isolates EEC function. Stimulated release should also be reported relative to basal release and to independently quantified EEC numbers. Chromogranin A-positive (CHGA⁺) cells provide a broad denominator, whereas tryptophan hydroxylase 1-positive (TPH1⁺) cells are more appropriate for 5-HT and hormone-defined populations for peptide-specific measurements (9, 10). Reporting both total output and output per relevant EEC separates reduced cell abundance from reduced average secretion per cell, provided viability and subtype composition are comparable. A pooled culture average still does not describe every individual cell. As with biopsy morphometry (18), these endpoints should be retained together rather than substituted for one another.

A third tier should confirm reproducible responses in 3D organoids with controlled access to the appropriate membrane surface, or in *ex vivo* mucosa from the relevant region. Only stimuli that discriminate donor groups across independent cultures, without barrier injury or dependence on extreme concentrations, should advance to human testing. The human challenge should preserve the selected stimulus class and delivery region as far as feasible, while measuring serial hormones alongside the prespecified motor or sensory outcome. Where sampling permits, organoids from the same participants can test whether their *in vivo* response is reproduced under controlled conditions and whether receptor perturbation alters it. This proposed back-translation would connect human physiology to epithelial mechanisms without assuming that either is sufficient alone.

These epithelial models do not reproduce enteric neurons, immune cells, resident microbiota, blood flow, or transit. Low baseline EEC yield can be addressed through NOTCH inhibition, inducible neurogenin-3 (NGN3/NEUROG3), and reporter-guided identification or purification; differentiation and enrichment can change subtype proportions, whereas a reporter chiefly makes selected cells accessible (9, 44, 46). Media, culture format, donor, and passage should be standardized or modeled explicitly, because culture conditions can substantially influence transcriptional profiles (46, 48). Strong responses to pharmacological or supraphysiological agonist concentrations do not by themselves justify a human nutrient challenge. Organoid abundance data are consequently most useful as a denominator and a guide to stimulus selection within a tiered human research programme.

## 6 Why do human IBS endocrine studies disagree?

Biological differences partly explain the apparent contradictions. Patients with different bowel-habit subtypes show different 5-HT responses (19, 20). Visceral sensitivity may define a more informative subgroup than diagnosis alone, as suggested by rectal EEC counts and PYY responses (17, 23). Sex can modify endocrine and sensory findings, while a rectal biopsy, jejunal biopsy, and systemic plasma measurement sample different compartments. Many foundational studies used Rome II or III criteria, so their case mix may not match current Rome IV populations (1, 3).

Measurement choices add another layer. Counting cells does not show how much they secrete. Molecular analyses have found altered mucosal 5-HT content and reduced 5-HT transporter expression even when enterochromaffin-cell populations were unchanged (33). Counts per high-power field and per unit mucosal area are not interchangeable (18). Fasting and stimulated measurements answer different questions, and a higher postprandial concentration may simply reflect a higher baseline, as shown clearly in IBS-D (20). Circulating concentrations also miss local paracrine and neurocrine signaling (8, 11).

Challenge protocols vary widely in energy content, macronutrient composition, route of administration, fasting duration, sampling schedule, and assay handling. For analytes such as 5-HT, how samples are collected, handled, and depleted of platelets strongly affects the measured concentration. Symptom timing matters as well: a hormone peak, a motor response, and a pain episode may occur on different timescales.

Future reports should state the Rome criteria and bowel-habit phenotype, define the challenge composition and route, provide the complete sampling schedule, distinguish absolute values from baseline-adjusted responses, and document assay handling. At least one objective physiological endpoint should be matched to the hypothesis. Researchers should prespecify sex, menstrual or hormonal status, diet, medication exposure, and visceral sensitivity when relevant. Standardization will not reveal a single IBS endocrine signature; it will make true subgroup differences easier to distinguish from methodological noise.

## 7 Translational implications and research priorities

### 7.1 Downstream receptor modulation

Pharmacological data show that endocrine pathways are modifiable, but they do not identify the upstream defect. Meta-analyses support clinical benefit from 5-HT3 antagonists in non-constipated IBS and IBS-D and from 5-HT4 agonism in constipation-predominant disease (35, 36). These responses confirm the importance of serotonergic signaling to motility, secretion, and symptoms. They cannot tell whether a given patient has altered EEC release, impaired uptake, abnormal receptor expression, or a downstream neural difference.

GLP-1 offers a second example, although the evidence is less mature. The short-acting analogue ROSE-010 improved acute IBS pain in a randomized placebo-controlled trial (37). Exploratory reanalysis suggested greater response in women and in IBS-C or mixed IBS, but nausea was more frequent at the higher dose (38). A 2025 meta-analysis supported short-term analgesic efficacy while emphasizing gastrointestinal adverse effects and the limited number of trials (39). These results relate to an acute experimental analogue and should not be extrapolated directly to long-acting metabolic GLP-1 receptor agonists.

### 7.2 Diet, GLP-1, and new observational signals

Recent studies illustrate both the promise and the interpretive difficulty of linking diet, hormones, and clinical outcomes. In an uncontrolled 12-week low-FODMAP intervention involving 30 patients with IBS-D or mixed IBS, circulating GLP-1 increased while symptom severity and body weight decreased (40). Without a control group, standardized meal challenge, or mechanistic measurements, the study cannot establish that the hormonal change mediated symptom improvement. It nevertheless shows that diet-related endocrine adaptation can be measured prospectively.

Large electronic-health-record analyses have also reported fewer coded gastrointestinal symptoms after initiation of GLP-1 receptor agonists in patients with IBS (41). The association is vulnerable to residual confounding, indication bias, and outcome-coding limitations. Real-world cohorts also document gastrointestinal symptom-related discontinuation (42). Together, these studies are hypothesis-generating; they do not establish long-acting GLP-1 receptor agonists as IBS therapy.

### 7.3 A practical research agenda

The most informative next step is not another cross-sectional measurement of a fasting hormone. Carefully phenotyped participants should undergo a defined meal or nutrient challenge with serial endocrine sampling, time-matched symptom recording, and an objective motor or sensory readout. Organoid screening should first guide the choice of stimulus and dose (Section 5.3 and Figure 2). Mucosal transcriptomics, receptor localization, and paired organoid or *ex vivo* secretion assays could then connect the *in vivo* response with cell state in a subset.

Local and neural processing cannot be read directly from a peripheral hormone curve. Site-matched biopsies can assess mucosal hormone content, transporters, degrading enzymes, and target-receptor localization; paired *ex vivo* stimulation can test local secretion and handling, but does not measure them continuously *in vivo* (19, 33). During the challenge, barostat sensory thresholds and motor recordings can quantify downstream responses (24, 27). In a mechanistic subset, a placebo-controlled crossover with a pathway-selective antagonist can test hormone-pathway involvement, and functional magnetic resonance imaging can assess central responses. A meal study in lean controls and patients with type 2 diabetes combined GLP-1 receptor blockade with serial blood sampling and functional imaging, but could not distinguish direct central from vagally mediated effects (49). This establishes methodological feasibility in humans rather than validation in IBS. These measurements therefore support converging inference, rather than direct proof of EEC-to-nerve transmission in humans.

Such studies should be large enough to prespecify bowel-habit subtype, PI-IBS status, sex, and visceral sensitivity. Repeated testing would establish whether response patterns are stable within a patient. The framework in Figure 1 separates five potential levels of dysfunction: luminal sensing, stimulus–secretion coupling, hormone metabolism or clearance, receptor responsiveness, and downstream neural or motor output. Similar symptoms could arise from abnormalities at different levels, which may explain why a single circulating biomarker has been elusive.

The challenge itself should be selected for the biological question. A mixed meal captures integrated physiology but makes it difficult to attribute a response to one nutrient class. An isolated oral nutrient or intraduodenal infusion offers greater mechanistic control, although it bypasses or alters cephalic, gastric, and mechanical components of eating. Crossover designs can compare nutrient classes within the same participant, provided energy load, osmolarity, volume, administration rate, fasting period, and medication restrictions are standardized. Symptoms should be recorded repeatedly rather than only at the end of the test, because sensory and endocrine peaks may not coincide.

Sampling and analysis also need to preserve dynamic information. Reports should present fasting values, absolute post-stimulus concentrations, incremental area under the curve, peak magnitude, and time to peak when appropriate; these endpoints cannot be substituted for one another, as the 5-HT literature demonstrates (19, 20). A repeat challenge in a subset would establish within-person reproducibility. Patient-derived organoids or *ex vivo* biopsy preparations could then test whether the *in vivo* pattern is accompanied by altered receptor expression or secretion under controlled stimulation. Exploratory clustering may be useful, but any response pattern should be confirmed in an independent cohort before it is described as a biological subgroup.

### 7.4 When could an endocrine response pattern become clinically useful?

An endocrine response pattern would need to clear a higher bar than statistical separation between IBS and control means. It should be reproducible within individuals, add information beyond bowel-habit subtype and symptom severity, correspond to a plausible cellular or physiological mechanism, and predict an objective outcome or treatment response in an independent cohort. Feasibility matters as well: a profile that requires prolonged sampling, invasive tissue collection, and several specialized assays may remain valuable for mechanism discovery without becoming a routine clinical test. The most realistic early use is likely enrichment of mechanistic trials—for example, identifying participants with a reproducible serotonergic handling pattern or an exaggerated lipid-evoked sensory response—rather than diagnosis of IBS itself.

Current evidence is well short of that threshold. Most studies are small, use different diagnostic eras, meals, assays, and tissue sites, and rarely repeat the same challenge. Stimulus-responsive profiling should therefore remain an experimental framework for locating pathway divergence and generating treatment hypotheses. Calling these patterns biomarkers or endotypes would be premature until stability, external validity, and clinical utility have been demonstrated.

## 8 Conclusions

IBS enteroendocrine research is better framed by asking how the intestinal sensory–endocrine system responds to a defined stimulus than by asking only how many EECs are present. Histological abnormalities remain relevant, especially in PI-IBS and other selected phenotypes, but cell counts alone cannot explain the diversity of postprandial symptoms. Human challenge studies show that endocrine findings vary with bowel habit, visceral sensitivity, sex, intestinal region, and the distinction between fasting, absolute fed, and baseline-adjusted responses.

Recent human organoid and transcriptomic data provide plausible mechanisms at several points between luminal detection and downstream neural or motor output. These cellular mechanisms have not yet been linked directly to endocrine response patterns in IBS participants. Standardized nutrient challenges that integrate endocrine, mucosal, motor, sensory, and symptom measurements offer a more coherent route to defining reproducible response patterns and testing whether they have therapeutic relevance.

## References

[1] Lacy BE Mearin F Chang L Chey WD Lembo AJ Simrén M. Bowel disorders. Gastroenterology. (2016) 150:1393–1407.e5. 10.1053/j.gastro.2016.02.031 27144627

[2] Black CJ Ford AC. Global burden of irritable bowel syndrome: trends, predictions and risk factors. Nat Rev Gastroenterol. Hepatol. (2020) 17:473–86. 10.1038/s41575-020-0286-8 32296140

[3] Nasser Y Shin A Ford AC Camilleri M Black CJ. Pathophysiology of irritable bowel syndrome. Lancet Gastroenterol. Hepatol. (2026) 11:1009–20. advance online publication. 10.1016/S2468-1253(26)00148-2 42462748

[4] Böhn L Störsrud S Törnblom H Bengtsson U Simrén M. Self-reported food-related gastrointestinal symptoms in IBS are common and associated with more severe symptoms and reduced quality of life. Am J Gastroenterol. (2013) 108:634–41. 10.1038/ajg.2013.105 23644955

[5] Van den Houte K Bercik P Simrén M Tack J Vanner S. Mechanisms underlying food-triggered symptoms in disorders of gut–brain interactions. Am J Gastroenterol. (2022) 117:937–46. 10.14309/ajg.0000000000001812 35506862 PMC9169752

[6] Hussein H Van Remoortel S Boeckxstaens GE. Irritable bowel syndrome: when food is a pain in the gut. Immunol Rev (2024) 326:102–16. 10.1111/imr.13374 39037230

[7] Gribble FM Reimann F. Function and mechanisms of enteroendocrine cells and gut hormones in metabolism. Nat Rev Endocrinol. (2019) 15:226–37. 10.1038/s41574-019-0168-8 30760847

[8] Nwako JG McCauley HA. Enteroendocrine cells regulate intestinal homeostasis and epithelial function. Mol Cell Endocrinol. (2024) 593:112339. 10.1016/j.mce.2024.112339 39111616 PMC11401774

[9] Smith CA Lu VB Bany Bakar R Miedzybrodzka E Davison A Goldspink D. Single-cell transcriptomics of human organoid-derived enteroendocrine cell populations from the small intestine. J Physiol. (2025) 603:7751–63. 10.1113/JP287463 39639676 PMC7617304

[10] Alcaino C Guccio N Miedzybrodzka EL Quale JR Lu T Davison A. Mechanisms of activation and serotonin release from human enterochromaffin cells. Cell Mol Gastroenterol Hepatol. (2025) 19:101610. 10.1016/j.jcmgh.2025.101610 40812684 PMC12547937

[11] Barton JR Londregan AK Alexander TD Entezari AA Covarrubias M Waldman SA. Enteroendocrine cell regulation of the gut–brain axis. Front Neurosci. (2023) 17:1272955. 10.3389/fnins.2023.1272955 38027512 PMC10662325

[12] El-Salhy M Gilja OH Gundersen D Hatlebakk JG Hausken T. Interaction between ingested nutrients and gut endocrine cells in patients with irritable bowel syndrome. Int J Mol Med. (2014) 34:363–71. 10.3892/ijmm.2014.1811 24939595 PMC4094590

[13] Grundeken E El Aidy S. Enteroendocrine cells: the gatekeepers of microbiome–gut–brain communication. NPJ Biofilms Microbiomes. (2025) 11:179. 10.1038/s41522-025-00810-x 40890205 PMC12402511

[14] Spiller RC Jenkins D Thornley JP Hebden JM Wright T Skinner M. Increased rectal mucosal enteroendocrine cells, T lymphocytes, and increased gut permeability following acute campylobacter enteritis and in post-dysenteric irritable bowel syndrome. Gut. (2000) 47:804–11. 10.1136/gut.47.6.804 11076879 PMC1728147

[15] Dunlop SP Jenkins D Neal KR Spiller RC. Relative importance of enterochromaffin cell hyperplasia, anxiety, and depression in postinfectious IBS. Gastroenterology. (2003) 125:1651–9. 10.1053/j.gastro.2003.09.028 14724817

[16] El-Salhy M Vaali K Dizdar V Hausken T. Abnormal small-intestinal endocrine cells in patients with irritable bowel syndrome. Dig Dis Sci. (2010) 55:3508–13. 10.1007/s10620-010-1169-6 20300845

[17] Park JH Rhee P- Kim G Lee JH Kim Y- Kim JJ. Enteroendocrine cell counts correlate with visceral hypersensitivity in patients with diarrhoea-predominant irritable bowel syndrome. Neurogastroenterol Motil. (2006) 18:539–46. 10.1111/j.1365-2982.2006.00771.x 16771769

[18] Mohammadian G Dlugosz A Shetye J Lindberg G. Quantification of mucosal EEC in jejunum: a comparative study of IBS patients and healthy controls. Scand J Gastroenterol. (2020) 55:543–8. 10.1080/00365521.2020.1764615 32442056

[19] Dunlop SP Coleman NS Blackshaw E Perkins AC Singh G Marsden CA. Abnormalities of 5-hydroxytryptamine metabolism in irritable bowel syndrome. Clin Gastroenterol Hepatol. (2005) 3:349–57. 10.1016/S1542-3565(04)00726-8 15822040

[20] Atkinson W Lockhart S Whorwell PJ Keevil B Houghton LA. Altered 5-hydroxytryptamine signaling in patients with constipation- and diarrhea-predominant irritable bowel syndrome. Gastroenterology. (2006) 130:34–43. 10.1053/j.gastro.2005.09.031 16401466

[21] Houghton LA Atkinson W Whitaker RP Whorwell PJ Rimmer MJ. Increased platelet depleted plasma 5-hydroxytryptamine concentration following meal ingestion in symptomatic female subjects with diarrhoea predominant irritable bowel syndrome. Gut. (2003) 52:663–70. 10.1136/gut.52.5.663 12692050 PMC1773651

[22] Sjölund K Ekman R Lindgren S Rehfeld JF. Disturbed motilin and cholecystokinin release in the irritable bowel syndrome. Scand J Gastroenterol. (1996) 31:1110–4. 10.3109/00365529609036895 8938905

[23] Van Der Veek PPJ Biemond I Masclee AAM. Proximal and distal gut hormone secretion in irritable bowel syndrome. Scand J Gastroenterol. (2006) 41:170–7. 10.1080/00365520500206210 16484122

[24] Furgała A Ciesielczyk K Przybylska-Feluś M Jabłoński K Gil K Zwolińska-Wcisło M. Postprandial effect of gastrointestinal hormones and gastric activity in patients with irritable bowel syndrome. Sci Rep. (2023) 13:9420. 10.1038/s41598-023-36445-1 PMC10256731 37296188

[25] Simrén M Agerforz P Björnsson ES Abrahamsson H. Nutrient-dependent enhancement of rectal sensitivity in irritable bowel syndrome. Neurogastroenterol Motil (2007) 19:20–9. 10.1111/j.1365-2982.2006.00849.x 17187585

[26] Simrén M Abrahamsson H Björnsson ES. Lipid-induced colonic hypersensitivity in the irritable bowel syndrome: the role of bowel habit, sex, and psychologic factors. Clin Gastroenterol Hepatol. (2007) 5:201–8. 10.1016/j.cgh.2006.09.032 17174611

[27] Grover M Berumen A Peters S Wei T Breen-Lyles M Harmsen WS. Intestinal chemosensitivity in irritable bowel syndrome associates with small intestinal TRPV channel expression. Aliment Pharmacol Ther. (2021) 54:1179–92. 10.1111/apt.16591 34472640

[28] Dontamsetti KD Pedrosa-Suarez LC Aktar R Peiris M. Sensing of luminal contents and downstream modulation of GI function. JGH Open. (2024) 8:e13083. 10.1002/jgh3.13083 38779131 PMC11109814

[29] Chao J Coleman RA Keating DJ Martin AM. Gut microbiome regulation of gut hormone secretion. Endocrinology. (2025) 166:bqaf004. 10.1210/endocr/bqaf004 40037297 PMC11879239

[30] Aguilera-Lizarraga J Florens MV Viola MF Jain P Decraecker L Appeltans I. Local immune response to food antigens drives meal-induced abdominal pain. Nature. (2021) 590:151–6. 10.1038/s41586-020-03118-2 33442055 PMC7610810

[31] Camilleri M Busciglio I Acosta A Shin A Carlson P Burton D. Effect of increased bile acid synthesis or fecal excretion in irritable bowel syndrome-diarrhea. Am J Gastroenterol. (2014) 109:1621–30. 10.1038/ajg.2014.215 25070056 PMC6994231

[32] Camilleri M Shin A Busciglio I Carlson P Acosta A Bharucha AE. Genetic variation in GPBAR1 predisposes to quantitative changes in colonic transit and bile acid excretion. Am J Physiol Gastrointest Liver Physiol. (2014) 307:G508–16. 10.1152/ajpgi.00178.2014 25012842 PMC4154122

[33] Coates MD Mahoney CR Linden DR Sampson JE Chen J Blaszyk H. Molecular defects in mucosal serotonin content and decreased serotonin reuptake transporter in ulcerative colitis and irritable bowel syndrome. Gastroenterology. (2004) 126:1657–64. 10.1053/j.gastro.2004.03.013 15188158

[34] Wei L Singh R Ghoshal UC. Enterochromaffin cells–gut microbiota crosstalk: underpinning the symptoms, pathogenesis, and pharmacotherapy in disorders of gut–brain interaction. J Neurogastroenterol Motil. (2022) 28:357–75. 10.5056/jnm22008 35719046 PMC9274469

[35] Ford AC Brandt LJ Young C Chey WD Foxx-Orenstein AE Moayyedi P. Efficacy of 5-HT3 antagonists and 5-HT4 agonists in irritable bowel syndrome: systematic review and meta-analysis. Am J Gastroenterol. (2009) 104:1831–43. 10.1038/ajg.2009.223 19471254

[36] Zheng Y Yu T Tang Y Xiong W Shen X Jiang L. Efficacy and safety of 5-hydroxytryptamine 3 receptor antagonists in irritable bowel syndrome: a systematic review and meta-analysis of randomized controlled trials. PLoS One. (2017) 12:e0172846. 10.1371/journal.pone.0172846 28291778 PMC5349445

[37] Hellström PM Hein J Bytzer P Björnsson E Kristensen J Schambye H. Clinical trial: the glucagon-like peptide-1 analogue ROSE-010 for management of acute pain in patients with irritable bowel syndrome: a randomized, placebo-controlled, double-blind study. Aliment Pharmacol Ther. (2009) 29:198–206. 10.1111/j.1365-2036.2008.03870.x 18945254

[38] Touny AA Kenny E Månsson M Webb DL Hellström PM. Pain relief and pain intensity response to GLP-1 receptor agonist ROSE-010 in irritable bowel syndrome: clinical study cross-analysis with respect to patient characteristics. Scand J Gastroenterol. (2022) 57:783–91. 10.1080/00365521.2022.2041084 35234561

[39] Mostafa MEA Alrasheed T. Improvement of irritable bowel syndrome with glucagon-like peptide-1 receptor agonists: a systematic review and meta-analysis. Front Endocrinol. (2025) 16:1548346. 10.3389/fendo.2025.1548346 PMC11932899 40134805

[40] Khan U Brønstad I Hillestad EMR Steinsvik EK Hausken T Berentsen B. Increase in circulating GLP-1 following low FODMAP diet in irritable bowel syndrome patients. Front Nutr. (2025) 12:1615671. 10.3389/fnut.2025.1615671 40880735 PMC12380553

[41] Khan SA Marasini A Shrestha A Bharadwaj HR Abdul RHM Dhali A. Impact of GLP-1 analogue therapy on gastrointestinal outcomes in patients with irritable bowel syndrome: a real-world TriNetX analysis. Dig Dis Sci. (2026) 71:5858–68. 10.1007/s10620-026-10171-w 42570075

[42] Gautam M Goel U Bader A Azim S Hassan N Sadeddin E. Patterns of prescription and discontinuation of glucagon-like peptide-1 receptor agonists among patients with irritable bowel syndrome. Ann Gastroenterol. (2025) 38:420–7. 10.20524/aog.2025.0971 40697433 PMC12277515

[43] Keszthelyi D Troost FJ Jonkers DM Van Eijk HM Dekker J Buurman WA. Visceral hypersensitivity in irritable bowel syndrome: evidence for involvement of serotonin metabolism—a preliminary study. Neurogastroenterol. Motil. (2015) 27:1127–37. 10.1111/nmo.12600 26031193

[44] Chang-Graham AL Danhof HA Engevik MA Tomaro-Duchesneau C Karandikar UC Estes MK. Human intestinal enteroids with inducible neurogenin-3 expression as a novel model of gut hormone secretion. Cell Mol. Gastroenterol. Hepatol. (2019) 8:209–29. 10.1016/j.jcmgh.2019.04.010 31029854 PMC6664234

[45] Roodsant T Navis M Aknouch I Renes IB Van Elburg RM Pajkrt D. A human 2D primary organoid-derived epithelial monolayer model to study host–pathogen interaction in the small intestine. Front Cell Infect Microbiol. (2020) 10:272. 10.3389/fcimb.2020.00272 32656095 PMC7326037

[46] Zeve D Stas E De Sousa Casal J Mannam P Qi W Yin X. Robust differentiation of human enteroendocrine cells from intestinal stem cells. Nat. Commun. (2022) 13:261. 10.1038/s41467-021-27901-5 35017529 PMC8752608

[47] Beumer J Geurts MH Geurts V Andersson-Rolf A Akkerman N Völlmy F. Description and functional validation of human enteroendocrine cell sensors. Science. (2024) 386:341–8. 10.1126/science.adl1460 39418382 PMC7616728

[48] Criss ZK Bhasin N Di Rienzi SC Rajan A Deans-Fielder K Swaminathan G. Drivers of transcriptional variance in human intestinal epithelial organoids. Physiol Genomics. (2021) 53:486–508. 10.1152/physiolgenomics.00061.2021 34612061 PMC8616596

[49] ten Kulve JS Veltman DJ Van Bloemendaal L Barkhof F Deacon CF Holst JJ. Endogenous GLP-1 mediates postprandial reductions in activation in central reward and satiety areas in patients with type 2 diabetes. Diabetologia. (2015) 58:2688–98. 10.1007/s00125-015-3754-x 26385462 PMC4630252

